# Impact of Shift Work and Long Working Hours on Worker Cognitive Functions: Current Evidence and Future Research Needs

**DOI:** 10.3390/ijerph18126540

**Published:** 2021-06-17

**Authors:** Veruscka Leso, Luca Fontana, Angela Caturano, Ilaria Vetrani, Mauro Fedele, Ivo Iavicoli

**Affiliations:** Department of Public Health, Section of Occupational Medicine, University of Naples Federico II, 80131 Naples, Italy; veruscka.leso@unina.it (V.L.); angela.caturano@live.it (A.C.); ilariavetrani20@gmail.com (I.V.); maurofedele85@gmail.com (M.F.); ivo.iavicoli@unina.it (I.I.)

**Keywords:** shift work, night work, long working hours, cognitive impairment, circadian rhythm, fatigue, sleep disturbances

## Abstract

Particular working conditions and/or organization of working time may cause important sleep disturbances that have been proposed to be predictive of cognitive decline. In this regard, circadian rhythm misalignment induced by exposure to night work or long working hours would be responsible for cognitive impairment. Nevertheless, evidence supporting this correlation is limited and several issues still need to be elucidated. In this regard, we conducted a systematic review to evaluate the association between shift/night work and cognitive impairment and address its main determinants. Information provided by the reviewed studies suggested that night work might have serious immediate negative effects especially on cognitive domains related to attention, memory and response inhibition. Furthermore, cognitive performance would progressively worsen over consecutive night shifts or following exposure to very long work shifts. Otherwise, conflicting results emerged regarding the possible etiological role that night work chronic exposure would have on cognitive impairment. Therefore, circadian rhythm desynchronization, lack of sleep and fatigue resulting from night work may negatively impact worker’s cognitive efficiency. However, in light of the considerable methodological variability of the reviewed studies, we proposed to develop a standardized research and evaluation strategy in order to obtain a better and comprehensive understanding of this topic.

## 1. Introduction

The term cognition refers to the internal mental processes that underlie how people perceive, remember, speak, think, make decisions, and solve problems [1]. In detail, the adoption of different and specific behaviors or actions in response to an external stimulus is regulated by several cognitive functions (also called domains) which are often activated simultaneously [2]. In this regard, it should be noted that, although several authors have tried to provide classifications of cognitive functions, a full consensus about their categorization is still lacking [3,4]. According to the fifth edition of the Diagnostic and Statistical Manual of Mental Disorders, the six main domains of cognitive function are defined as follows, complex attention, executive function, learning and memory, language, perceptual-motor function, and social cognition [5]. Therefore, it is evident that correct functioning of these mental processes is absolutely essential in order to ensure a normal social life that guarantees the possibility of interacting appropriately with other people and the surrounding environment [6]. On the other hand, the deterioration of cognitive performance, as well as a reduction, even temporary, of its efficiency can also have serious consequences on working life, especially in carrying out hazardous/safety-sensitive working activities. Indeed, in order to be carried out in complete safety conditions, they require the constant keeping of the highest standards regarding reaction times, attention, vigilance and concentration [7,8].

In this regard, it is well-acknowledged that a decline in neurocognitive functioning is associated with advancing adult age, even if, in many cases, the observed aging-related neurocognitive impairment does not affect the everyday domains of social and occupational life as negatively as one would suppose [9,10,11]. Increasing age is considered the strongest risk factor for cognitive decline, but also other parameters may play an important role in its occurrence. For instance, having a specific apolipoprotein E genotype (APOEε4) is a well-established genetic risk factor, since its prevalence is higher in subjects affected by mild cognitive impairment with respect to the general population [12,13,14,15] and it has been correlated with a more rapid rate of global cognitive decline and deterioration [16]. Moreover, it should be pointed out that there is sufficient evidence to support the correlation between several medical (e.g., diabetes, obesity, hypertension, depression, hypercholesterolemia) and social or lifestyle risk factors (e.g., smoking, excess alcohol consumption, lack of physical exercise and sedentary lifestyle, low social and cognitive engagement, low education level) and cognitive impairment [17,18].

Interestingly, a number of studies suggested that sleep disturbance is predictive of cognitive decline in older people and in those with neurodegenerative disorders and such problems should be identified and treated early to prevent a deterioration of cognitive functions [19,20]. Indeed, several studies conducted under laboratory settings, investigating the potential effects of total or partial sleep deprivation on cognition, showed a wide range of cognitive alterations [7,21]. For example, lack of sleep decreased ability to concentrate or learn and remember new information, reduced alertness/preparedness levels or executive function and slowed reaction time [8]. In this regard, some particular working conditions and/or organization of working time may cause important sleep disturbances. In detail, working atypical shifts, outside the normal daylight hours (7/8 am–5/6 pm), which can encompass early-morning, late evening or night shifts [22] might lead to the shift work sleep disorder that is a condition characterized by trouble sleeping, excessive sleepiness and fatigue [23]. Furthermore, shift workers, especially those who carry out night work, experience a reduction of sleep of about two hours [24]. Then, it is plausible to hypothesize that shift work, through the disturbing effect exerted on sleep, might potentially play a negative role in workers’ cognitive performance. In other words, sleep should be considered a possible mediator of the adverse effects induced on cognitive functions by working conditions [7].

In this regard, recent numerous studies suggested that shift work (particularly that including working at night) and in some cases also long working hours (that is working 41 or more hours per week) are able to cause important modifications of cognitive functions [21,25,26,27]. In detail, both qualitative and quantitative sleep disturbances (insomnia, shortened sleep, frequent sleep interruptions, irregular sleep cycle) associated with the shift work would induce a circadian rhythm misalignment which, in turn, activating neuroendocrine system and affecting specific brain areas would be ultimately responsible for the observed cognitive impairment [19,25,28]. However, available findings of the possible correlation between shift work exposure and negative effects on cognitive performance are still scarce and sometimes controversial [27]. Indeed, although different studies have shown adverse effects of shift work on mental processes, several issues, such as which cognitive domains are most affected and to what extent, the identification of pathophysiological mechanisms underlying the observed effects, the assessment of a potential dose-response relationship, the possibility of functional recovery after leaving shift work and the acknowledgment of the impact correlated to chronic exposure, still need to be clarified. In this context, this systematic review aimed to comprehensively evaluate the causal relationship between the performance of shift work (including night work) and the occurrence of cognitive impairment in order to provide useful information to deepen knowledge on this topic and address future research towards the aforementioned unresolved critical issues.

## 2. Materials and Methods

**Figure 1:**
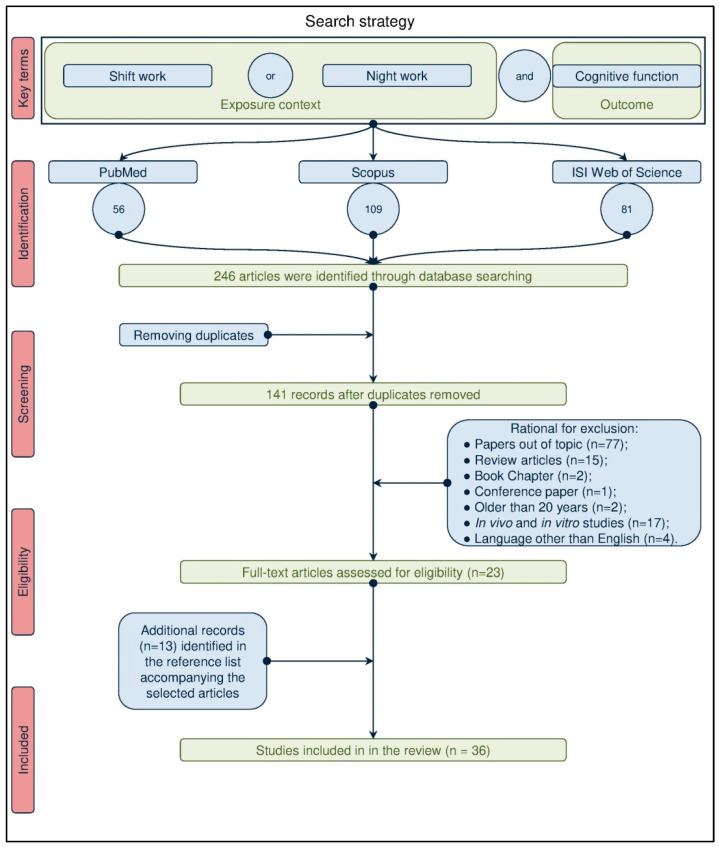
Flowchart of the search strategy used to identify studies of interest.

We carried out a systematic advanced literature search according to the Preferred Reporting Items for Systematic Reviews and Meta-Analyses Statement (PRISMA) criteria [29] to retrieve studies that investigated the potential relationship between exposure to shift work and occurrence of cognitive detriment. The research work has been conducted until 19 January 2021 on the three main scientific databases, PubMed, Scopus, and ISI Web of Science. The search strategy used the wordings “shift work”, “night work”, “long working hours” combined through the “AND” Boolean operator with “cognitive impairment” as key terms to contextualize the exposure and identify the outcome of the investigation, respectively (Figure 1).

Three of the authors independently evaluated all the titles and abstracts provided by the computerized search and then they picked out eligible studies in the review accordingly to the inclusion criteria. With regard to the latter, we included different types of human peer-reviewed research articles, including descriptive epidemiological and/or occupational surveys, cross-sectional, cohort, case-control and -series studies, that were published in English language and explored the potential impact of atypical work schedules on the performance of different cognitive domains. The citation pool of publications to be included in this review was further supplemented by analyzing the reference list accompanying the selected articles. Differently, we excluded reviews, notes, book chapters, letters, editorials, conference papers, experimental studies on laboratory animals and humans simulating night shifts as well as articles published in languages other than English and more generally any study that did not have the specific purpose of investigating the adverse effects on cognitive functions caused by performing shift work.

## 3. Results

The first step of the search strategy conducted on PubMed, Scopus, and ISI Web of Science databases, consisting in identifying the articles of interest for review, has retrieved 56, 109 and 81 records respectively, thus accounting for a total of 246 articles. Subsequently, after eliminating the duplicates, from the 141 remaining articles we excluded, accordingly to the defined inclusion criteria, 118 studies since 77 were considered off-topic based on the analysis of title and abstract, 18 were reviews articles, book chapters or conference paper (15, 2 and 1, respectively), 17 were experimental studies conducted on cellular or animal models and four were published in languages other than English. Therefore, we ultimately identified 23 articles as potentially eligible in the review. The full texts of these studies were obtained and a critical evaluation of the manuscripts was then carried out. This analysis allowed to further enlarge the citation pool of relevant publications retrieving 13 additional articles of interest that were identified in the reference lists accompanying the initial 23 selected articles. Overall, our search retrieved a total of 36 articles suitable for review (Figure 1).

The overwhelming majority of these investigations were conducted in Europe and Asia (16 and 11 studies, respectively), seven studies were carried out in North America and only two came from other continents (Africa and Australia). The number of workers studied were quite heterogeneous ranging from 10 flight nurses recruited in the study of Thomas et al. [30] to 7143 current and former shift workers included in the study of Titova et al. [31]. Most studies used self-administered questionnaires to obtain data on socio-demographic characteristics, anthropometric data, educational level, sleep patterns and duration, comorbidities, smoking habits, alcohol and caffeine consumption, working history, atypical work schedule (shift work type and duration of shift work), psychosocial risk factors, and occupational exposures. The different cognitive functions were evaluated using several standardized and validated neuropsychological tests (Table 1). Moreover, in some studies, electrophysiological measures by electroencephalography and the determination in biological matrices of different hormones such as growth hormone (GH), cortisol and its precursors, prolactin (PRL), thyroid-stimulating hormone (TSH) and melatonin were also carried out in order to analyze the possible physio-pathological mechanisms underlying the observed alterations of cognitive domains. Importantly, in these studies, the potential role of different parameters that are recognized risk factors for the cognitive decline (e.g., age, family history for dementia and/or related diseases, hypertension, cerebral vasculopathies, metabolic syndrome, disorders of sleep and mood, physical inactivity, social isolation, lower education level and psychosocial factors) and that could represent confounding factors in the interpretation of the results have been often verified, albeit in quite different ways and combinations [7,8,21,25,26,27,30,31,32,33,34,35,36,37,38,39,40,41,42,43,44,45,46,47,48,49,50,51,52,53,54,55,56,57,58,59]. Finally, most of the articles have studied the short-term effects of shift work, while few studies have analyzed the possible impact of long-term chronic exposure on cognition [27,31,46,47,48,49,50,51].

The following paragraphs summarize information concerning the possible association between shift/night work and cognitive alterations in involved workers according to different exposure conditions and lengths of shift works obtained from the papers selected for the review.

### 3.1. Short-Term Effects Induced by Shift and Night Work on Cognitive Functions

Several studies evaluated the short-term consequences induced by shift work on different cognitive functions assessing the cognitive performance immediately after the end of the shift both following a single or consecutive days of shift works (Table 2).

In the healthcare setting, anesthesia residents engaged in a night shift of 12 h demonstrated a greater deterioration in cognitive functions, i.e., attention, learning and memory compared to their day shift colleagues [32]. Memory impairment was also observed in 13 emergency physicians both after a daylight and an overnight shift [33]. Concerning hormonal patterns, in night workers, a delay or reduction in morning cortisol spikes was evident. Similar results were observed in healthcare workers in whom the scores of several neuropsychological tests (carried out immediately after a night shift) resulted significantly lower compared to colleagues who worked the daylight shift [36]. Interestingly, in this study, the poorer cognitive performance on attention–concentration, recognition, and long-term memory was significantly correlated with higher levels of oxidative stress markers such as total antioxidant capacity and total oxidant status [36].

In line with these results, a group of 100 shift nurses showed lower scores in general intellectual ability, mental speed, response inhibition, attention, simple reaction time, and working memory tests, after performing a night shift versus a day shift. Such impairment might be due to the qualitative-quantitative alterations of sleep that, altering the sleep–wake rhythm, could affect the frontal and prefrontal cortex executive functions [37]. In the same professional group Esmaily et al. [25], administering different cognitive tests to workers before and after different work shifts (i.e., 07:30 to 14:00, 14:00 to 19:30 and 19:30 to 07:30), found out that nurse’s working memory and interference score were significantly reduced at the end of each work shift, although with the largest decrease after a night shift rather than morning and evening shifts [25]. This suggests that, apart from the shift fatigue, night work has a key role in affecting cognitive functions [25].

A study, conducted on workers belonging to different productive sectors and industries, proved evidence that an atypical work schedule on the previous day might have a negative impact on cognitive efficiency in the following day (especially for verbal memory and selective attention) [7]. These detrimental effects were independent of the participant’s sleep characteristics, other working conditions and age. Additionally, the potential negative impact of night shift work on cognition was further confirmed also in employees of the business process outsourcing (BPO) sector [34,35]. Indeed, compared to non-shift controls, night workers of both genders obtained poorer scores in neuropsychological tests for learning, memory and response inhibition, whereas mental speed and visual working memory were affected only in male and female workers, respectively.

In contrast Petru et al. [38] in an automobile production plant, showed no differences in cognitive functions or psychomotor abilities of day shift workers compared to permanent night workers. Moreover, in both worker groups, comparable results were obtained at the cognitive tests performed at the beginning and at the end of the shift, while improvements were detected in concentration and accuracy assessment. Similarly, maritime pilots did not show objective cognitive deficits compared to controls even if some subjective disturbances in specific cognitive domains were observed [39]. Howbeit, these findings should be treated with caution, due to the small size of the recruited cohort, and the lack of a unique definition for shift duration and distribution whose irregularity was primarily related to the workload and the number and type of ships that arrived. Consequently, to define a causal correlation between the work shift features, i.e., type and length, and the possible adverse impact on cognitive functions is quite a challenging issue. For this reason, the authors refer more properly to shift work-related sleep disruption [39].

In the context of the short-term effects, it is noteworthy pointing out that several studies have also investigated the impact of exposure to consecutive night shifts on cognitive domains (Table 2). In this regard, Rollinson et al. [40], in 12 medicine interns in the Emergency Department found a significant decrease in visual memory capacity over attention and vigilance comparing values obtained at the beginning and at the end of the first and third of four consecutive night shifts. Comparably, a progressive (especially after three nights) and significant worsening in the detection and identification tasks were determined at the end of night shift week in nine anesthetic trainees, while no effects were detected during the day shift period [41]. A lower cognitive performance, particularly in perceptual and motor ability, was demonstrated in fast rotating nurses performing two consecutive night shifts with respect to those who worked four consecutive night shifts [42]. When the authors attempted to correlate cognitive alterations and changes in the levels of different sleep-related hormones (e.g., growth hormone, cortisol, prolactin and TSH), they could point out that nurses in fast rotating patterns showed attentional and learning impairments and higher prolactin blood levels compared to nurses who performed four consecutive nights, whereas no significant changes of growth hormone and cortisol levels were observed [43]. 

In the petrochemical industry, control room operators experienced a significant decline in working memory, sustained attention, and reaction time both at the end of day and night shifts lasting 12 h, although with a greater impairment after these latter ones [8,21]. This was ascribed to the fatigue induced by long working hours per shift and the relatively short resting time, but also to the fast rotation from day to night shifts responsible for a lack of biological adaptation to the night work. In line with this statement, workers engaged in seven consecutive nights demonstrated better cognitive performance indexes compared to those involved in four night-shift patterns, suggesting a more effective circadian rhythm adaptation [8,21,44]. Moreover, in seven consecutive night workers the maximum peak phase of salivary melatonin, an acknowledged indicator of night shift adaptability, was detected at the end of the shift whereas in the other group it was reached around 3:00 am [21]. However, the limited number of enrolled subjects (*n* = 60) and the multiple factors that may affect the adaptation of circadian rhythms to the shift pattern, i.e., light exposure, environmental conditions, shift work features, as well as individuals’ characteristics (i.e., chronotype), require caution for a correct interpretation of the consecutive shift role. In this regard, Ferguson et al. [45] on miners working in a seven-day/seven-night shift pattern, observed that reaction times were significantly decreased at the end of both shifts and across consecutive shifts. The variation in the time of melatonin onset was minimal, although significant. This suggested a lack of true adaptation of the circadian rhythm, as the melatonin rhythm remained synchronized with the daily light/dark cycle rather than shifting later as expected in the case of adaptation to the work/rest rhythm. Similarly, the lack of an adaptation when running consecutive night shifts would also be confirmed by the results of Griffiths et al. [41] that proved no evidence of slowing of deterioration after performing several consecutive night shifts.

### 3.2. Long-Term Exposure to Shift and Night Work and Effects on Cognitive Functions

Apart from the short-term and “sub-acute” effects (due to the execution of a single night shift or several consecutive night shifts, respectively) an extremely interesting topic of research concerns the potential impact on cognitive domains deriving from a prolonged exposure for many years (Table 3).

In this regard, a lower cognitive efficiency (in terms of immediate free recall) was determined in male salaried workers exposed to shift work at the time of the neuropsychological tests [46]. Interestingly, a significant association was observed between the length of employment in shift work and the cognitive function with poorer memory performance in workers exposed for 10–20 years compared to those exposed for 1–4 years, regardless of age and self-reported sleep quality. This may represent a cumulative, reversible effect of chronic exposure to shift work on the disruption of circadian rhythms, as it was not observed in workers who had ceased exposure for more than four years [46]. Similarly, a possible adverse cognitive effect reversibility has been proposed by Marquie et al. [47]. The authors, in fact, found comparable cognitive performance scores in day workers and former shift workers who had left shift work for at least five years, and notably, these values were better than those observed in current shift workers. Furthermore, shift work was correlated to a chronic decline of cognitive functions that became significant when the exposure duration exceeded 10 years [47]. The same time frame to recover was also reported by Titova et al. [31], who demonstrated comparable cognitive function scores in former shift workers (who had quit shift work for >5 years) and non-shift workers. Importantly, regardless of age and sleep characteristics, poorer cognitive performance was observed in current and recent former shift workers with respect to non-shift workers.

However, the negative relationship between the long-term exposure to shift work and the detrimental impact on cognitive domains has not been confirmed in several other studies. For example, in older female adults (≥70 years of age), with a mild-life history of exposure to shift work for different time periods, no association could be determined between the engagement in rotating night-shift work and the average cognitive status at an older age or the rate of cognitive decline over time [48]. Comparably, midlife exposure to shift work or night work was reported to be not associated with significant cognitive change on verbal, spatial and memory abilities, processing speed and general cognitive function in late-life and no significant differences in cognitive aging could be observed between workers exposed to shift/night schedule and typical day shift workers [27]. In healthcare and administrative staff, no evidence for an association of shift work with cognitive level and change in cognitive function was reported by Weinmann et al. [49]. When the potential interaction between long-term sleep loss, induced by prolonged and sustained exposure (≥25 years) to irregular work shifts, and post-retirement cognitive decline or dementia was evaluated in male maritime pilots, cognitive scores resulted in a normal range and no relationship emerged with the number of working years [50]. These results were confirmed in a subsequent study, carried out in a smaller sample of the same professional category by the same group of researchers both after a work week and a rest week [51]. In this population, no cerebral amyloid-β deposition, whose increase is associated with sleep loss, could be detected.

### 3.3. Effects of Long Working Hours on Cognitive Functions

Regardless of the pattern of shift work, another element that could significantly impact the workers’ cognitive functions is the excessive length of the work shift, generally longer than 8 h each, that may overall be responsible for long working hours in a week that is usually categorized in three analytical categories of 41–48, 49–54, and ≥55 h/week [60] (Table 4). This is a relevant issue considering the hazardous potential of the longest category (≥55 h) in inducing adverse health effects [60]. In detail, long shifts, night works and long working hours in a week would result in a combined action of both circadian rhythms alterations and increased levels of fatigue experienced which, in turn, could more severely affect the cognitive efficiency of workers. 

This may be the case of interns of emergency departments, anesthesia residents and control room operators who were engaged in 12 hours’ shifts. Fatigue, resulting from a very long work shift, may be responsible for the deterioration of cognitive functions, particularly reaction times, reported by control room operators after day and night shifts [32]. In other studies, where the workload and tiredness were similar between different work schedules, the observed decline in cognitive functions would be more related to the desynchronization of circadian rhythm than to the fatigue.

Obviously, the detrimental effects induced by long working hours on cognitive domains may be closely related to the duration of the shift and therefore, the longer the working time, the greater the magnitude of the effect. In this regard, among medical intensive and coronary care unit interns, the rate of attentional failures during night work was more than doubled in the healthcare workers who followed the traditional shift schedule (with extended work shifts of 30 consecutive hours) compared to the intervention one (in which work shifts were a maximum of 16 consecutive hours) [52]. However, not all cognitive functions were equally affected, as demonstrated in anesthesiology residents, before and after a 24 h shift [53]. In fact, a significant impairment of cognitive efficiency in carrying out long-term and monotone tasks were described after the shift, whereas concentration and working memory were not damaged [53]. Similarly, hospital physicians who performed extended, continuous day-night shifts (24 h) showed a significantly greater decline of neurocognitive abilities and mental status, i.e., mood, vigilance, agitation and visual memory, compared to colleagues in day shifts [54]. Interestingly, cognitive alterations determined in continuous day-night shift workers were significantly correlated to a reduction in urinary pregnanetriol and androsterone/etiocholanolone ratio levels [54]. Comparable results were obtained by Adams and Venter [55] in anesthesiology trainees who showed an important decline of reaction time in psychomotor and attention cognitive functions at the end of 14 h shifts. Deterioration of cognitive efficiency following extended shifts work was also proved in paramedics and firefighters who worked 12 h and 24 h shifts, respectively [56]. Similarly, the cognitive efficiency of firefighters (assigned to different rotations of extended 15 and 24 h work shifts, including night) was significantly affected by the exposure to night work that was associated with lower scores of composite memory, verbal and visual memory, complex attention, psychomotor and motor speed [26].

In a recent study, adults over the age of 65 years who worked ≥ 40 h/week, compared to those who worked < 40 h, were more likely to have decreased cognitive performance. Older people who took prolonged daytime naps (>45 min), compared with those who did not take naps, were more likely to have a decreased cognitive performance [57]. In junior and senior anesthetists, tests addressing recognition, motor and total reaction time, critical flicker fusion, peripheral recognition time, and response measure failed to show significant differences before and after 24 h shift on-call duties [58]. However, an age-focused analysis demonstrated that senior anesthetists had a tendency towards a prolonged reaction time. In the prospective study by Thomas et al. [30] the amount of daily sleep and cognitive performance were compared in flight nurses who worked 12 h in the evening versus 18-h shifts during a 72-h duty period. The authors showed that, despite the significant decline in daily sleep during both service programs, no significant drop was observed in before and after cognitive test scores. Long hours worked were reported to have a negative effect on sleep patterns. Nurses who worked 8 h a day had significantly greater sleep efficiency and less drowsiness upon awakening than those who worked 12 h on rotation, who, conversely, were significantly more sleepy [59]. 

However, the different experimental designs adopted in the above-revised studies, the heterogeneous work shift schedules and the variable occupational fields explored, make the interpretation of the role of homeostatic and circadian factors in affective cognitive performance a quite challenging issue requiring deeper additional research. 

## 4. Discussion

The 24-h modern society is characterized by extremely pressing social and economic needs which, in order to be satisfied, have led over the years to a profound reorganization of work cycles, thus increasing the presence of shift and night work in numerous production sectors. Atypical working time is particularly common in healthcare, the transportation industry and hospitality and, according to the sixth European Working Conditions Survey, the percentage of workers in shift work increased significantly from 17% in 2010 to 21% in 2015 [61]. Unfortunately, shift work and night work have been correlated with several negative health and well-being outcomes such as cardiovascular diseases, gastrointestinal and metabolic disorders and to a lesser extent with cancer, mental health problems (e.g., anxiety, depression, dementia) and reproduction-related complaints (e.g., increased risk of miscarriage, low birth weight and premature birth) [24,62]. Furthermore, in particular, the night work would seem to be one of the main causes that contribute to increasing the likelihood of accidents at work [7]. In this regard, a recent systematic review has evaluated the strength of the association between exposure to shift and/or night work and risk of dementia pointing out that further research is needed to verify and establish the presence of a causal relationship [28]. However, in this perspective, these results underline the importance of increasing our level of knowledge regarding the potential negative impact exerted by atypical working time on the mental state and more generally on cognitive functions. Indeed, from the point of view of occupational medicine, it is not only important to identify the possible occurrence of mental disorders in workers exposed to shift work but also to define fitness for work of subjects who carry out hazardous/safety-sensitive working activities requiring a high level of cognitive performance since, in these cases, the maintaining of an efficient and reactive cognition is an essential requirement to work in complete safety conditions.

In this context, the findings of this review demonstrated that shift work (particularly night shifts) has serious immediate negative effects on cognitive functions, especially regarding the cognitive domains related to attention, memory and response inhibition (Table 2). The most important contributing factors to this cognitive impairment observed in shift workers would be represented by disruption of circadian rhythm, sleep deprivation and fatigue. The desynchronization of the endogenous timekeeping system, resulting from working and sleeping at the wrong circadian phase, would determine in shift workers a greater vulnerability to cognitive deficits [63,64,65], since the circadian rhythm that regulates sleep and wakefulness would be adversely affected by an external stimulus such as shift/night work or long and erratic working hours [66,67,68]. This internal biological timekeeping system is evolutionarily conserved in order to function as well as possible during the day and to facilitate sleep at night [69]. The suprachiasmatic nucleus located in the hypothalamus is the master clock regulating circadian rhythms in humans and its activity is mainly driven by environmental and humoral signals [70]. Indeed, light inputs and melatonin secretion (during the day and night, respectively) are responsible for the daily reset of the circadian rhythms [71]. In particular, regarding the pineal gland melatonin secretion it increases two hours before usual bedtime remaining sustained throughout the night when its circulating levels exert an inhibitory action on the suprachiasmatic nucleus [70,71]. Therefore, in shift workers, the misalignment between behavioral cycles and the endogenous timekeeping system would be responsible for the loss of stable phase relation of the circadian rhythm of different physiological variables such as sleep/wake cycle, the rhythmic secretion of melatonin, or the core body temperature (that is inversely related to melatonin levels) and likely this loss would be one of the main determinants of the vulnerability to cognitive deficits. Indeed, in this regard, it is noteworthy to underline that a phase delay of the rest-activity rhythm has been suggested as a risk factor for developing mild cognitive impairment, whereas a phase advance in the melatonin secretion has been observed in patients suffering from this condition [72,73,74]. Moreover, it should be noted that physiological aging has been associated with a progressive deterioration of circadian rhythms (e.g., increased fragmentation, decreased amplitude, phase advance of the rest-activity and temperature rhythms, modifications of the sleep-wake structure) and, in turn, these alterations have been correlated with a progressive decline of cognitive functions [75].

In addition, also the qualitative and quantitative alterations of sleep might play a significant role in determining a lower cognitive efficiency since it has been proved that night workers sleep an average of two hours less than workers with a fixed day shift and have also a poorer quality of sleep due to negative influences on the second and rapid eye movement stages of sleep [45,76,77]. Moreover, another factor that could impact the workers’ cognitive performance, especially working memory and alertness, is fatigue which is often objectively greater during extended and/or night shifts [78,79]. The recognition of the specific weight that each of these parameters plays in causing cognitive decline would be desirable especially in order to define and apply the most appropriate corrective prevention and protection measures. However, it should be considered that, due to the organization of the shift systems and schedules, the aforementioned causal factors are concomitant and interdependent and their effects are difficult to distinguish as they often tend to overlap each other. For example, in most studies that evaluated the short-term effects of night work (Table 2), the administration of neuropsychological tests took place on the first day of the shift schedule (which often involved multiple and consecutive night shifts), immediately at the end of the night shift. Therefore, it is quite intuitive that the negative effects observed in these workers are largely attributable to the fatigue due to having just finished the night shift, while the contribution of both the repeated circadian rhythm desynchronization and the sleep deprivation is difficult to evaluate. Then, although these results are important in demonstrating the ability of night work to affect some specific cognitive domains, they are limited in preventive terms as they represent a snapshot but do not inform about the evolutionary aspects (in the short and medium period), of cognitive decline related to the repeated misalignment of circadian clock or to the progressive lack of sleep. Therefore, in order to verify a potential cumulative effect due to these two causal factors, it would be interesting to be able to compare the results of the cognitive tests administered at the beginning (after a rest period) and at the end of each work shift within the shift schedule or, at least, compare the scores of tests performed at the end of the first night shift with those made at the end of the last night shift of the rotation cycle.

Indeed, some studies included in this review (Table 2) have used such a methodological approach by repeating the neuropsychological tests at different time points within the shift schedule. For example, Rollins et al. [40] administered the cognitive battery tests at the beginning and at the end of shift on the first and third of four consecutive night shifts, whereas Griffiths et al. [41] carried out the neuropsychological assessment before and after seven consecutive night shifts of 12 h duration. Furthermore, some authors have also compared cognitive data of several shift worker groups engaged in different rotation systems that provided for the execution of a variable number of consecutive night shifts [8,21,42,43,44]. These studies further confirm that night work has a considerable adverse effect on some neuropsychological functions such as attention and memory, but, in this regard, the interesting data relates to the fact that the information obtained suggests that cognitive performance progressively deteriorates over consecutive night shifts. In this regard, it is noteworthy to underline that these studies also provide useful data in terms of possible preventative measures that could be applied to minimize the observed negative effects. In fact, it should be noted that has been reported the possibility for circadian rhythms to be fitted to night shifts but the speed and rate of this adaptation remain to be determined [80,81,82]. Overall, Chang et al. [42,43] and Kazemi et al. [8,21] supported the idea that a slow rotation system allows a better adaptation of circadian rhythms than a fast rotation system ensuring workers an adequate amount of time to adjust their biological clock to the night shift. In this regard, the improvement of cognitive functions observed with an increasing number of consecutive nights might be explained in the light of the participants’ circadian rhythm adaptability. Indeed, subjects working more consecutive night shifts are more likely to adapt their circadian rhythm to the new situation than those who spend a smaller number of night shifts at work. This hypothesis would also be sustained by the data concerning the levels of melatonin detected in two different groups of workers subjected to a fast or slow shift system [21]. In fact, although no significant difference was observed between the two groups in terms of salivary melatonin concentrations and sleepiness indices, the significant effect of time x shift interaction indicates that circadian rhythm follows a comparatively more disorderly pattern among fast rotating shift workers (four consecutive night shifts). In participants working seven consecutive night shifts there was a steady increase in the melatonin rhythm and sleepiness (that reached their peak at the end of the shift), whereas when considering four consecutive night shift workers the melatonin rhythm (such as sleepiness trend) experienced ups and downs during the shift reaching its maximum around 3:00 am, that is a clear indicator of circadian rhythm disorder [21]. Therefore, circadian misalignment is present in both situations since the expected phase relation of sleep/wake and melatonin rhythm has been lost for workers belonging to both groups, even though the melatonin rhythm is more regular in workers experiencing seven night shifts with respect to four night shifts. Nevertheless, more evidence is needed to exactly determine the optimal number of night shifts for achieving circadian rhythm adaptability and pinpoint the adaptability rate since other studies have previously suggested that a fast rotation schedule would be more effective in reducing the disruption to body rhythms [83].

In many workplaces, the duration of the night shift usually exceeds the canonical eight working hours. Indeed, most studies investigating the immediate effects of shift and night work or the cumulative effect of consecutive night shifts (Table 2) have recruited workers who performed extended shifts (usually 12 h in duration). Therefore, in these cases, the possible etiological role of fatigue (related to long working hours) in causing cognitive damage but also the extent of its impact on cognitive performance are difficult to identify as they are strictly interconnected with changes in the circadian rhythm and/or sleep deprivation. However, fatigue certainly plays an important role in contributing to poorer cognition in shift workers, as proved by some studies that compared the cognitive performance of workers scheduled on shifts of different lengths (Table 4). These results showed that, usually, workers exposed to very long work shifts (i.e., 30 or 24 h) had significantly poorer cognitive performance than their colleagues on shorter work shifts (i.e., 8, 12, 15 or 16 h), even if some studies provided different findings [30,58,59], suggesting the need of further research to explore the interaction between fatigue and cognitive impairment. In this regard, if the aim is to evaluate the contributing role of fatigue in the occurrence of possible cognitive disorders according to the different length of work shifts, it is essential that workers are as much as possible comparable to each other, not only as regards sociodemographic characteristics but, also with regard to occupational risk factors that in some way could affect the level of fatigue (e.g., night work, features of work activities, workload). Unfortunately, in the field studies, this is rather challenging due to multiple organizational, technical and procedural difficulties that are connected to the work cycle and its related needs. Consequently, when this is not entirely possible, it is essential to have available valid and reliable methodological tools to measure the level of fatigue experienced by workers. The studies included in this review, which assessed the degree of fatigue, used quite different approaches, employing in some cases tests that provided an objective measure of this parameter and in other self-administered questionnaires that supplied a subjective evaluation. In principle, in order to avoid the self-report bias, it would be desirable to use investigation methods that can provide objective data such as the simple reaction time test that, showing a good level of reliability, has been validated for assessing fatigue (prolonged and/or slowed reaction times are suggestive of an increased level of fatigue) [84].

With regard to the hypothetical causal association between chronic exposure to shift and/or night work and the occurrence of long-term effects on cognitive functioning, the information from the reviewed studies is somewhat conflicting (Table 3). Indeed, Rouch et al. [46], Marquie et al. [47] and Titova et al. [31] showed that a shift and/or night work history is significantly correlated to poorer neuropsychological performance and, this association would be both dose-dependent (stronger association was observed for exposure lasting between 10 and 20 years) and reversible (similar findings were detected between former shift workers, who had stopped shift work for more than four or five years, and non-shift workers). On the contrary, Bokenberger et al. [27] and Devore et al. [48] did not support the hypothesis that shift work history can induce long-term effects on cognition since the neuropsychological test scores of shift workers and non-shift workers did not differ significantly. A plausible reason for explaining these discordant results would lie in the fact that these epidemiological studies (conducted on large cohorts of workers) were not designed to specifically address the possible relationship between shift or night work and cognitive impairments. Consequently, the subjects recruited in these studies belonged to an extremely wide range of occupations and economic sectors in which presumably the work activities carried out and the related occupational risk factors, but also and more specifically, the organization of shift work, the speed of rotation and the hours of work shifts were widely different. Moreover, the analysis of the main characteristics of shift work were rather incomplete, being usually based on fairly general questions on whether “the job involved shiftwork with changing schedules”, “job often involved going to bed after midnight (more than 50 days per year)”, “the total number of years during which you worked rotating night shifts (at least three nights/month in addition to days or evenings in that month)?”. Then, a real and thorough analysis of the shift work organizational mode regarding for example the duration of shifts, the total number and the number of consecutive night shifts, or shift rotation was missing. Therefore, this issue must be further studied by designing and carrying out studies on homogeneous categories of workers, possibly exposed to shift work for several years, and in which the different aspects of this particular organization of working time are evaluated in a rigorous and detailed manner. This assessment appears particularly urgent and desirable, especially considering that the retirement age of workers is progressively increasing and that therefore the potential negative impact of shift work on their cognitive functions could have a synergistic and additive effect with the normal aging of the nervous system.

In this perspective, it seems appropriate to pay attention to some methodological considerations, since comparing the results of the studies that have dealt with this topic is often a complex and challenging task. In this regard, the need to conduct a careful analysis of the main shift work determinants (that is hypothesized to have a role in inducing adverse effects on cognitive domains) has just been discussed, as we have already mentioned the opportunity to recruit and compare groups of workers with overlapping occupational and socio-demographic characteristics and also to analyze differences by gender and chronotype in order to minimize the possible influences of confounding factors. However, it is also important to highlight that the reviewed studies have evaluated the cognitive functions using a wide range of validated tests and questionnaires which, moreover, were administered in different ways and times. Therefore, in order to facilitate the comparison between these results and to obtain a better and comprehensive understanding of this topic, it would be useful to define the main cornerstones of a standardized research and evaluation strategy that might represent a sort of gold standard of reference.

## 5. Conclusions

Timely recognition of neurocognitive impairment is essential in optimizing prevention strategies and treatment options [20]. However, to get an early diagnosis of this condition it is necessary to know what to look for and above all where to look which means, in other words, that it is fundamental to have a comprehensive knowledge of the risk factors associated with cognitive decline, in such a way as to be able to design, apply and implement appropriate screening programs. In this regard, it is well-recognized that increasing age is the most important risk factor for cognitive detriment but, it is also widely accepted that, besides this unchangeable risk factor, this condition can be influenced by other modifiable risk factors such as medical conditions and lifestyle habits. In this context, several recent epidemiological studies have suggested the hypothesis that psychophysical consequences deriving from the performance of shift work may play an important role in determining a worsening of the cognitive functions of exposed workers. From the point of view of occupational medicine, this is a particularly interesting topic both for the high number of workers who experience these forms of atypical working time and for the prolonged exposure that in many cases affects the entire working life of people. However, at the same time, this topic also deserves attention for the possible public health implications since mild cognitive impairment is considered to be a transitional stage between the expected cognitive decline of normal aging and dementia and these two pathological conditions share most of the currently known risk factors [85].

In this regard, information obtained from the papers selected for the review as a whole suggests that the desynchronization of circadian rhythms, the lack of sleep and fatigue resulting from the performance of shift and night work can negatively impact the cognitive efficiency of workers. This decrease in cognitive performance, especially as regards the domains of attention and memory, can represent an extremely serious issue particularly in those worker categories who carried out hazardous/safety-sensitive working activities which require the constant keeping of the highest standards regarding reaction times, attention, vigilance and concentration. In this regard, our findings provide rather interesting information to implement in the workplaces effective countermeasures that might be able to reduce the negative effects exerted by shift and night work on cognitive functions. In detail, a better organization of shift work that takes into account the opportunity to reduce working hours (especially at night), take advantage of slow rather than fast rotation systems and ensure adequate rest periods between work shifts, could limit the cognitive impairment, thus contributing to improving the psychophysical well-being of workers.

## Figures and Tables

**Table 1:** Neuropsychological tests used in the studies included in the review and relative cognitive function explored.

Neuropsychological Test	Cognitive Domain Assessed
Attention network test (ANT)	Alerting, orienting, and executive control
Auditory Verbal Learning Test (AVLT)	Attention, memory and learning ability in the auditory and verbal domain
Central nervous system vital signs (CNSVS)	Composite memory, verbal memory, visual memory, complex attention, psychomotor speed, motor speed, processing speed, reaction time, cognitive flexibility, executive functioning, and neurocognitive index.
Chalder Fatigue Questionnaire (CFQ)	Physical and mental fatigue
CogStateTM computerized test battery	Simple reaction (detection), simple decision making (identification), immediate memory, monitoring, learning
Cognitive Failure Questionnaire (CFQ)	Burden of daily subjective cognitive errors
Conner’s Continuous performance Test (CCPT)	Attention
Continuous Performance Test (CPT)	Attention, vigilance
Critical flicker fusion	Arousal and individual integrative capacity
d2 test	Alertness, capacity to deal with stress and capacity of concentration
Delayed Recognition Span Test (DRST)	Visual memory capacity
Digit Span subtest (subset of the Wechsler Adult Intelligence Scale—WAIS)	Working memory and executive function
Digit–symbol substitution test (DSST) (Subtest of the Wechsler Adult Intelligence Scale—WAIS)	Motor speed, attention, and visuo-perceptual functions
Digit vigilance test	Attention
Doors Test	Overnight memory consolidation
Early dementia questionnaire	Signs of early dementia
East Boston Memory Test	Verbal memory
Executive functioning test	Executive functions
Letter and semantic fluency, Boston Naming Test	Semantic memory, language
Letter-Number (LN) span	Working memory, audible memory, and attention
Logical Memory test (Subtest of the Wechsler Adult Intelligence Scale—WAIS)	Episodic memory
Maintenance of Wakefulness Test	Ability to stay awake and alert during the day
Maslach Burnout Inventory (MBI-D)	Features of burnout
Mental speed test	Mental speed and flexibility
Montreal Cognitive Assessment (MoCA)	Visuospatial function, naming, memory recall, attention, language, abstraction, delayed recall and orientation
Multitasking test (MTT)	Ability to ignore task-irrelevant information, as part of executive function
N back test (verbal and visual)	Auditory and visual working memory
One card learning test	Visual memory
One-touch stockings of Cambridge (OTS)	Spatial planning and working memory as part of executive function
Paced Auditory Serial Addition Test (PASAT)	Sustained and divided attention and rate of information processing
Paired associates learning (PAL)	Visual episodic memory
Mood Questionnaire (MDBF)	Mood, vigilance, and agitation
Rapid visual information processing (RVP)	Response sensitivity under time pressure
Reaction time (RTI)	Psychomotor speed (ability to attend and respond to a critical stimulus)
Repeatable Battery for the Assessment of Neuropsychological Status (RBANS)	Immediate and delayed memory, language, attention, and visuospatial memory
Response inhibition	Executive function: control and coordination of cognitive skills like analytical thinking, working memory, planning, cognitive flexibility
Rey Auditory Verbal Learning Test (RAVLT)	Semantic memory and language
Santa Ana Form Board Test.	Psychomotor speed, coordination
Selective attention test (subtest of the Sternberg test)	Selective attention
Simple reaction time test (SRT)	Processing speed and motor control
Spatial working memory (SWM)	Visuospatial working memory
State-Trait Anxiety Inventory (STAI)	State and trait anxiety
Stroop Color and Word Test (SCWT)	Cognitive processing, cognitive flexibility, resistance to interference from outside stimuli, and ability to cope with cognitive stress
Symbol Searching Test (SST) (Subtest of the Wechsler Adult Intelligence Scale—WAIS)	Cognitive, perceptual, and motor ability
Taiwan University Attention Test (TUAT)	Attention processing
Telephone Interview of Cognitive Status (TICS) (a telephone version of the Mini-Mental State Examination)	Orientation, concentration, short-term memory, language, praxis, and mathematical skills
Test of Attentional Performance (TAP 2.0)	Attention
Test of Neuropsychological Malingering (TNM)	Attention
Trail Making Test (A–B)	Motor speed and attention
University of Southern California Repeatable Episodic Memory Test (REMT)	Immediate memory span, new learning, recognition memory, and susceptibility to interference
Verbal Fluency test (VFT)	Language (number of words generated in 1 min)
Vienna test system	Capabilities essential for the driver’s license
Visual analog scale (VAS) for alertness	Subjective alertness
Visual recognition test	Visual memory
Visual attention tasks (VAT) (Subtest of the Wechsler Adult Intelligence Scale–WAIS)	Cognitive, perceptual, and motor ability.
Visual Aural Digit Span Test (VADST)	Awareness, attention, concentration, short term memory
Visual recognition test (FVW)	Visual memory
Vocabulary Test	Verbal intelligence and speech comprehension
Wechsler Adult Intelligence Scale Fourth edition (WAIS-IV)	Working memory and executive function
Wechsler Memory Scale-Revised (WMS-R)	Attention–concentration, visual and verbal memory, delayed recall, mental control, general memory
Wisconsin Card Sorting Test (WCST)	Abilities of abstract reasoning and of changing cognitive strategies as environmental circumstances change, measure of frontal lobe ability
Working memory test	Executive function includes control and coordination of cognitive skills like analytical thinking, working memory, planning, cognitive flexibility

**Table 2:** Studies addressing short-term effects induced by shift work and single or consecutive night shifts on cognitive functions.

Study Location	Subjects	Characteristics of Shift and/or Night Work	Cognitive Testing	Results	Quality Rating According to NOS	References
USA	12 Emergency physicians (6 M, 6 F; mean age = 28 years).	Work shift schedule: 07–19 for day shift and 19–07 for night shift. 4 consecutive day shifts, 2 days off, 4 serial night shifts and then 2 nights off. Length of shifts: 12 h.	Delayed Recognition Span Test (DRST); Continuous Performance Test (CPT); Santa Ana Form Board Test. Administration: at the beginning of a day shift and at the beginning and end of night shifts 1 and 3 of 4 consecutive night shifts.	DRST: significant (18.5%) decrease in visual memory capacity from the beginning to the end of the shift. CPT or the Santa Ana Form Board Test: no significant differences between the beginning and end of the shift.	Satisfactory	Rollinson et al., 2003 [40]
Turkey	33 Anaesthesia residents: 15 in daytime (5 M, 10 F; mean age = 29 ± 2.5 years) and 18 in night shifts (4 M, 14 F; mean age = 29 ± 2.4 years).	Work shift schedule: 08–20 for day shift and 20–08 for night shift for five days. Length of shifts: 12 h.	State-Trait Anxiety Inventory (STAI); Auditory Verbal Learning Test (AVLT); Visual Aural Digit Span Test (VADST). Administration: before and after shift in the first day of 5 days’ period.	AVLT: Scores decreased significantly in both groups after the shifts but the impairment in learning was more evident in the post-shift evaluation of night workers. VADST: Poorer scores were observed in night workers after the shifts, whereas no significant differences were detected in the pre- and post-shift assessment of day shift workers. State anxiety levels were similar in both worker groups or before and after the shifts.	Satisfactory	Saricaoglu et al., 2005 [32]
Germany	44 male automobile workers: 20 in rotating day shifts (mean age = 38.7 ± 11.9 years; continuous employment on this type of shift = 14.9 ± 2.7 years) and 24 in night shifts (mean age = 38.9 ± 9.2 years; continuous employment on this type of shift = 7.5 ± 5.9 years).	Work shift schedule: 06:00–14:30 for early shifts, 14:30–22:30 for late shifts and 22:30–06:00 for night shifts for five days. Length of shifts: 8–8.30 h.	Morningness—Eveningness Questionnaire (MEQ); Visual analog scale (VAS) for subjective alertness; d2 test; Vienna test system for reaction time and the alertness, visual alertness, attention deficits, reaction speed, and the relative capacity to deal with stress. Administration: before (during the first 90 min) and after shift (during the last 90 min).	Cognitive and psychomotor testing carried out before and after the shift provides overlapping findings in day and night workers; In both groups, cognitive performance improved during the shift; No effect on cognitive performance was exerted by the chronobiological type and age in combination with shift schedule.	Satisfactory	Petru et al., 2005 [38]
Fitzory, Victoria, Australia	9 anesthetic trainees	Work shift schedule: 08–18 for day shift and 19.30–7.30 for night shift. Length of shifts: from 10 to 12 h for day shift; 12 h for night shift.	CogStateTM computerized test battery. Administration: before and after seven consecutive day shifts and seven consecutive night shifts.	No significant change in performance before or after any day shift, nor at the commencement of each night shift. Speed of performance for detection and identification tasks: significant deterioration at the end of the night shift as the week progressed. A significant decline in cognitive performance was determined in anesthetic registrars after a series of night shifts.	Satisfactory	Griffiths et al., 2006 [41]
France	2337 workers present and former wage earners covering a wide range of occupations and economic sectors (1152 M; 1185 F).	Work shift schedule: before 6 am or after 10 pm (*n =* 258 workers). Length of shifts: <8 h (*n =* 1511); 8–10 h (*n =* 669); >10 h (*n =* 157).	Memory test adapted from the Rey auditory verbal learning test (RAVLT); Digit–symbol substitution test, a sub-test of the Wechsler adult intelligence scale (WAIS); Selective attention test derived from the Sternberg test. Administration: between 8:00 and 19:00.	An atypical work schedule on the day before was significantly associated with poorer cognitive performance in immediate free recall and in delayed free recall and selective attention. No interaction between age and working hours (working before 6 am or after 10 pm).	Very good	Ansiau et al., 2008 [7]
Taiwan	62 Nurses (62 F; mean age = 26.4 ± 2 years).	Work shift schedule: rotation on two (*n* = 21), three (*n* = 20), and four (*n* = 21) consecutive night shifts.	State-Trait Anxiety Inventory (STAI); Stanford Sleepiness Scale (SSS); Wisconsin Card Sorting Test (WCST); Taiwan University Attention Test (TUAT); Digit Symbol Substitution Test (DSST); Symbol Searching Test (SST). Administration: 3–4 am on the last night shift of the rotation.	No significant differences in STAI scores, and SSS scores, as well as in WCST and TUAT parameters between the three groups. DSST and SST: significant reduction in subjects who worked two consecutive night shifts compared with those who worked four consecutive night shifts.	Unsatisfactory	Chang et al., 2011 [42]
Australia	29 Mining operators (Mean age = 37.4 ± 6.8 years).	Work shift schedule: 05.45–18.00 for day shift and 17.45 to 06.00 for night shift. Length of shift:12 h.	Reaction time test. Administration: at the beginning and at the end of shift.	Reaction times in psychomotor performance: significantly slowed at the end of both night and day shifts and across consecutive shifts.	Good	Ferguson et al., 2012 [45]
USA	13 Emergency physicians (EPs) (9 M; 5 F; mean age = 38.2 ± 7.0 years).	Work shift schedule: Day shifts per month (mean = 7.1 ± 3.4). Overnight shifts per month (mean = 2.5 ± 1.4). Length of shifts: 6–8 h.	Paced Auditory Serial Addition Test (PASAT); University of Southern California Repeatable Episodic Memory Test (REMT); Trail Making Test (TMT); Stroop Color-Word Test Pittsburgh Sleep Quality Index (PSQI); Chalder Fatigue Questionnaire (CFQ). Administration: 30 min before and immediately after at least one daylight shift and at least one overnight shift.	REMT: significant decrease in the number of recalled words after overnight (27 ± 6.2) and daylight shifts (28.9 ± 5.0) compared to preshift (31.6 ± 5.2 and 31.3 ± 5.2, respectively). Yes–no intrusions: significant increase after day shifts (0.3 ± 0.8) compared to pre-shift (0.2 ± 0.5). Stroop Color-Word Test: significant decrease in the test score between pre- (111.2 ± 2.4) and post-overnight shift (106.8 ± 10.7). PASAT and TMT: no significant changes between pre- and post-shifts.	Satisfactory	Machi et al., 2012 [33]
India	50 Business process outsourcing (BPO) employees (50 M; mean age = 29.14 ± 2.96 years) exposed to regular shift. 50 non-BPO employees non-shift workers (mean age = 28.52 ± 2.77 years).	BPO employees worked on computers for at least 5 h a day or 25 h a week. Length of shifts: 11–12 h per day and at times 14 h.	Digit symbol substitution test (DSST); Digit vigilance test; Auditory verbal learning test; Stroop test; N back test (verbal and Visual). Administration: in a fixed order, one right after the other, during a single session in a quiet room at the end of work.	DSST significant increase in BPO employees (197.18 ± 39.70) compared to controls (180.22 ± 19.78). Learning and memory score: significantly lower scores in BPO employees (31.56 ± 4.86 and 30.80 ± 3.29, respectively) compared to controls (37.40 ± 4.61 and 34.64 ± 3.43, respectively). Stroop test: significantly higher scores in BPO employees (170.34 ± 43.78) compared to controls (136.26 ± 17.78).	Satisfactory	Shwetha et Sudhakar 2012 [34]
Turkey	90 Health care workers (44 M; 46 F; mean age = 27.59 ± 4.41 years). 45 night-shift workers and 45 daytime shift workers.	Work shift schedule: rotated between 08:00–16:00 and 16:00–08:00 shifts for 3-week intervals. Individuals in the night shift group worked 3 days a week and participants in the daytime group worked 5 days a week. Length of shifts: 8 h for day shifts and 16 h for night shifts.	Wechsler Memory Scale-Revised (WMS-R); Auditory Verbal Learning Test (AVLT); Stroop Color-Word test (SCWT). Administration: at 08:00.	Cognitive performance: daytime working staff scored significantly higher than night shift workers in the verbal memory, attention concentration, and the digit span, forward sub-scales of WMS-R, as well as on AVLT while had a significantly lower score in the consistency of recall.	Good	Ozdemir et al., 2013 [36]
Taiwan	59 Nurses (59 F); 23 off-duty: (OD group) mean age = 26.1 ± 1.9 years); 20 working 2 consecutive night shifts: (2 NS group) mean age = 26.0 ± 2.0 years); 16 working 4 consecutive night shifts: (4 NS group) mean age = 27.1 ± 2.0 years.	Work shift schedule: repetitive blocks of two consecutive day shifts (8–16 or 08–17.30), two evening shifts (16–12), two-night shifts (12–08), and then at least 1 day off. Length of shifts: 8–9.30 h for day shifts and 20 h for night shifts.	Maintenance of Wakefulness Test; State-Trait Anxiety Inventory; Stanford Sleepiness Scale; Visual attention tasks (VAT); Wisconsin Card Sorting Test (WCST); Modified Multiple Sleep Latency Test.	No significant differences among the groups in WCST performance. Performance of perceptual and motor tasks, as measured by the DSST and SST, was better in the OD group than in either night shift group. Although all groups showed a trend toward improved perceptual and motor abilities during the daytime, the improvements were more significant in the OD group and the 4 NS group than in the 2 NS group.	Good	Chang et al., 2014 [43]
India	50 Regular shifts BPO employees (50 F; mean age = 27.82 ± 2.36 years). 50 Controls non-BPO employees not working in shift (50 F mean age = 28.58 ± 2.42 years).	See Shwetha et Sudhakar 2012	Auditory Verbal Learning Test (AVLT); Stroop Color-Word Test (SCWT); Verbal N Back test; Visual N Back test. Administration: in a fixed order, one right after the other, during a single session in a quiet room at the end of work.	BPO employees performed poorly compared to their controls in tests for learning and memory, response inhibition and visual working memory. No changes between groups in tests for verbal working memory. AVLT: learning and memory scores were significantly lower in BPO employees (33.48 ± 4.49 and 32.24 ± 3.93, respectively) than in controls (36.76 ± 4.79 and 35.84 ± 3.77, respectively).	Satisfactory	Shwetha et Sudhakar 2014 [35]
Iran	60 Petrochemical control room shift workers (60 M; mean age = 30.1 ± 2.46 years).	Work shift schedule: 7 consecutive night, or day shifts, and 7 consecutive days off. 07- 19 for day shift; 19- 07 for night shift. Length of shifts: 12 h.	Continuous performance test (CPT); N-back test; Reaction time (RTI). Administration: at the end of both day and night shifts.	N-back—score: significant decrease of at the end of the night (94.6 ± 6.3) and day (102 ± 6.74) shifts compared to the beginning (104.8 ± 8.1 and 106.1 ± 7.9, respectively); Response time: significant increase of at the end of night (730.5 ± 79.4) and day (729.5 ± 87) shifts compared to the beginning (663 ± 102 and 687.3 ± 104.5, respectively); Reaction time: significant slowdown at the end of the night (244.4 ± 34.7) and day (254.8 ± 38.5) shifts compared to the beginning (227.5 ± 28.4 and 227.6 ± 28.3, respectively); Commission error in CPT test: significant increase of at the end of the night (1.55 ± 1.13) and day (0.95 ± 0.83) shifts compared to the beginning (0.68 ± 0.66 and 0.4 ± 0.56, respectively).	Satisfactory	Kazemi et al., 2016 [8]
Iran	60 control room operators (CROs) in the largest petrochemical com- plex divided in two groups: 30 CROs (mean age = 29.2 ± 1.9 years) in 4 consecutive night-shifts; 30 CROs (mean age = 31.1 ± 2.6 years) in 7 consecutive night-shifts.	Work shift schedule: the day shift was 07–19 for the day shift; 19–07 for the night shift.Length of shifts: 12 h	Continuous performance test (CPT); Simple reaction time test (SRTI). Administration: in the first and the last 30 min at the beginning and the end of the shifts.	The number of consecutive night shifts had a significant impact on commission errors and reaction time but not a significant effect on the N-back score, response time to the N-back test, omission errors and response time in the CPT test. Correct answers and working memory response times decreased significantly while intentional errors and sleepiness increased during shift work.	Good	Haidarimoghadam et al., 2017 [44]
India	100 Nurses (97 F; 3 M; mean age = 25.06 years).	Work shift schedule: staff was posted to day shift for one month, and to night shift for the next one month. Length of shifts: 8-h for six days a week.	Montreal Cognitive assessment (MoCA); Simple reaction time (SRTI); Executive function: response inhibition and working memory. Administration: during the end of day shift and 3–4 days after start of the night shift.	MoCA test: significantly lower score in night compared to day shift workers. Execution and memory test scores: significantly higher during day compared to night shifts. SRT was significantly quicker during the day than at night shifts.	Satisfactory	Kaliyaperumal et al., 2017 [37]
Iran	60 Petrochemical control room shift workers (60 M; mean age = 30.2 ± 2.0 years).	Work shift schedule: 19–07 for night shifts. One group (4N7D3N7O): 4 nights, 7 days, 3 nights, 7 days off for one group; One group (7N7D7O): 7 nights, 7 days, 7 days off. Length of shifts: 12 h.	Continous performance test (CPT); N- back test. Administration: at the beginning, in the middle and at the end during the shift.	Working memory (N-back test): the mean number of correct responses was significantly higher in the 7 compared to the 4 consecutive night shift groups. Sustained attention (CPT): The mean number of omission errors and response time were significantly higher in the 4 than 7 consecutive night shift groups.	Satisfactory	Kazemi et al., 2018 [21]
Iran	35 F Nurses (range age = 25–40 years).	Work shift schedule: two morning shifts (7:30 to 14), two evening shifts (14 to 19:30), two night shifts (19:30 to 7:30) and one day rest. Length of shifts: 6. 30 h for morning ed evening shifts and 12 h for night shifts.	Digit Span subtest (Wechsler Adult Intelligence Scale); Stroop Color-Word Test (SCWT). Administration: at the beginning and the end of working shifts in three shifts (morning, afternoon and night). An interval of 3 days was considered for performing after-shift test to eliminate the learning effect.	Working memory score: significant decrease at the end of all three shifts (morning: 7.74 ± 0.92 before vs. 7.37 ± 0.81 after); (evening: 7.40 ± 0.81 before vs. 6.97 ± 0.95 after; *p* = 0.002) (night 7 ± 1.11 before vs. 5.83 ± 1.07 after). Interference score: significant decrease after evening (1.03 ± 1.62 before vs. 1.60 ± 1.59 after) and night shift (0.94 ± 1.62 before vs. 5.14 ± 1.92 after). Response time: no significant changes during one shift.	Unsatisfactory	Esmaily et al., 2020 [25]
Netherlands	20 Maritime pilots (median age = 43.6 ± 4.36 years). 20 Controls (median age = 36.5 ± 5.71 years).	Work shift schedule: 24 hr for seven consecutive days followed by a week off. Length of shifts. 24 h.	Cognitive Failure Questionnaire (CFQ); Reaction time (RTI); Spatial working memory (SWM); Paired associates learning (PAL); Rapid visual information processing (RVP); Multitasking test (MTT); One-touch stockings of Cambridge (OTS). Administration: the first day off after a work week	CFQ: significant overall decrease in maritime pilots (22.5) compared to controls (34); a significant increase in confusion in maritime pilots (6.9 ± 3.24) compared to controls (4.65 ± 2.80); a significant increase in social confusion in maritime pilots (6.3 ± 2.60) compared to controls (5.1 ± 2.49); Significant increase in names and words in maritime pilots (7.5) compared to controls (5); no significant changes in orientation score. No significant differences between maritime pilots and controls on RTI RVP, SWM, PAL, MTT and OTS tests.	Satisfactory	Thomas et al., 2020 [39]

**Table 3:** Long-term effects induced by shift work on cognitive functions.

Study Location	Subjects	Length of Employment in Shift Works-Characteristics of the Shift Worker Groups	Cognitive Testing	Results	Quality Rating According to NOS	References
France	3237 workers (1660 M; 1577 F)	Shift-workers: current (265 M; 321 F); former (346 M, 242 F); never (1049 M; 1014 F).	Memory test adapted from the Rey Auditory Verbal Learning Test (RAVLT); Digit Symbol Substitution Test (DSST); Selective attention test derived from the Sternberg test.	Current male shift-workers showed significantly lower cognitive performance in terms of immediate and delayed free recalls, speed measures, compared to never exposed workers. Memory performance: significantly decreased in men according to the 10–20 year shift-work duration). Memory scores were significantly higher for women who stopped 4 years before.	Good	Rouch et al., 2005 [46]
United States	Female nurses: (*n =* 16,190; mean age = 74.3 ± 2.3 years).	Shift-work experience duration: 1–9 years (*n =* 7685); 10–19 years (*n =* 1341); ≥20 years (*n =* 1028); No shift workers: 6136.	Telephone Interview of Cognitive Status (TICS); Immediate recall and delayed recall of the East Boston Memory Test. Administration: subsequently repeated 3 times, at 2-year intervals.	Mean differences in average cognition were similar for both global and verbal scores in nurses with ≥20 years of shift-work history and no-shift nurses, while a significantly lower cognitive status was assessed through the TICS with respect to no-shift nurses.	Very good	Devore et al., 2003 [48]
France	3119 present and former wage earners covering a wide range of occupations and economic sectors. Shift work current or past exposure: *n* = 1484; No-shift workers: *n* = 1635.	Current shift workers *(n* = 381); ≤5 years recency (*n* = 417); >5 years recency (*n* = 295). Rotating shift workers: 1- 10 years (*n* = 583); ≥10 years (*n* = 534).	Verbal episodic memory test; Digit-Symbol Substitution test (DSST); Selective attention test derived from the Sternberg test. Administration: the first measurement (t1, 1996), and 5 (t2) and 10 (t3) years later.	Cognitive performance: significantly lower in shift workers vs. controls; global, 56.0 ± 10.71 vs. 53.3 ± 10.60; memory, 50.8 ± 10.61 vs. 48.5 ± 10.46; speed: 78.5 ± 8.77 vs. 76.5 ± 9.05. Rotating shift workers: significantly lower cognitive performance in >10 years workers compared to those engaged for 1–10 years: global, 51.8 ± 10.49 vs. 55.4 ± 10.08; memory, 47 ± 10.23 vs. 50.3 ± 10.33; speed, 75.6 ± 8.84 vs. 78.1 ± 8.2.	Very good	Marquie et al., 2015 [47]
Sweden (2015)	7143 Partecipants (age range = 45–75 years).	Non shift-work (*n* = 4611); past shift-work (*n* = 1531); recent former shift-work (*n* = 358); current shift work (*n* = 643).	Trail Making Test (TMT A-B). Administration: during the visit to 1 of 2 Swedish test centers.	No significant differences in performance were found in TMT between past and non-shift workers. Current and recent former shift workers required more time to complete the TMT test compared to no-shift workers. The ratio between two subsets of TMT tests, as a more accurate measure of executive functions, was significantly higher in current and recent former shift workers compared to non-shift ones.	Very good	Titova et al., 2016 [31]
Sweden (1986–2002)	Cohort from the Swedish Adoption Twin Study of Aging (SATSA) 1986: *n* = 595; 299 M, 296 F; mean age = 62.6 ± 8.5 years). Cohort from the Screening Across the Lifespan Twin (SALT) sample (*n* = 320).	Shift workers (*n* = 106); years in shift work: 1–9 (40.6%); 10–19 (30.2%); ≥20 (29.2%). SALT-sample: night shift workers.	Verbal ability test; Memory tests. Administration: measures of cognitive performance were assessed in 9 waves during 1986–2012.	Midlife shift work was not associated with mean cognitive performance at retirement age or with a rate of cognitive change during the 27-year follow-up period for any of the cognitive factors (verbal, spatial, memory, processing speed, and general cognitive ability).	Very good	Bokenberger et al., 2017 [27]
Germany(January–April 2017)	500 Healthcare workers (389 F, 111 M; mean age = 61.6 ± 4.2 years). 75 completing a socio- demographic questionnaire; 47 subjects participating in cognitive testing (*n* = 47; 11% of the total sample).	Mean duration of shift-work experience: 29 and 24 years in any shift system and in a system including night shifts, respectively.	Repeatable Battery for the Assessment of Neuropsychological Status (RBANS); German version of the Trail Making Test (TMT); Letter-Number (LN) span; Vocabulary Test.	Cognitive test scores: in the Vocabulary Test none of the subjects scored below average, in the TMT test (participants’ cognitive performance speed) 17% had a score below average; in the RBANS test (neuropsychological status) 6% were below average. Clinical evaluation: 17 participants showed slight difficulties in at least one subscale of the cognitive tests. The frequency of indication for slight or pronounced impairment did not differ between those working with and without shifts.	Good	Weinmann et al., 2018 [49]
Netherlands	50 Retired maritime pilots (mean age = 71.7 ± 7.7 years).	History of >25 years of work on irregular schedules.	Cognitive Failure Questionnaire (CFQ); Early Dementia Questionnaire (EDQ); Pittsburgh Sleep Quality Index (PSQI); Sleep-wake diaries; QoL (EQ-5D); Hospital Anxiety and Depression Scale (HADS). Administration: participants were scheduled for 1 visit at which they supplied answers to 6 questionnaires.	CFQ: all scores were within the normal range. The highest sub-score was observed on the confusion subscale and the lowest on the orientation subscale. EDQ: all participants remained below the median cut-off median (8), suggesting that participants did not show signs of early dementia.	Good	Thomas et al., 2019 [50]
Netherlands	19 Maritime pilots (median age 53 ± 3.4 years); 16 Controls (mean age = 57 ± 2.9 years).	Work history: an average of 20 years (mean = 19.8; range 10 to 30 years).	Logical Memory Subtest (WMS-IV LM); Rey Auditory Verbal Learning Test (RAVLT)); Letter and semantic fluency, Boston Naming Test; Digit Span subtest (WAIS-IV); Trail Making Test (TMT A-B); WAIS-IV Coding; Test of Attentional Performance (TAP 2.0); Doors Test. Administration: performed in the morning following polysomnography (in 2016 and 2017).	No difference between maritime pilots and controls on episodic memory tests. Small differences in semantic memory and language with slightly better performance for maritime pilots than controls. Working memory, executive function and attention: no significant differences between the groups. All test scores were within normal ranges adjusted for age and education based on available normative data.	Satisfactory	Thomas et al., 2020 [51]

**Table 4:** Studies addressing the effects of long working hours on cognitive functions.

Study Location	Subjects	Characteristics of Shift and/or Night Work	Cognitive Testing	Results	Quality Rating According to NOS	References
USA	20 Interns in intensive care units (mean age = 28.0 ± 2.0 years).	Two three-week rotations on two work schedules: a traditional schedule with extended duration work shifts of ~30 consecutive hours every other shift (~77–81 h/week); an intervention schedule with a maximum of 16 consecutive hours for single shift and of ~60–63 h/week.	Continuous electrooculography was used to assess attentional failures defined as an intrusion of slow-rolling eye movements into polysomnographically confirmed episodes of wakefulness during work hours.	Interns had significantly less than half the rate of attentional failures while working during the night (from 11 p.m. to 7 a.m.) compared to a traditional schedule.	Good	Lockley et al., 2004 [52]
Salt Lake City, Utah.	10 life Flight nurses (3 M; 7 F; mean age = 39.9 ± 4.3 years).	Work shift schedule: two different duty schedules: three consecutive 12-h evening shifts (19–07). The second program in two 18-h shifts (07–01) separated by a 24-h rest period.	Rey auditory Verbal Learning Test (RAVLT); Conner’s Continuous Performance Test (CCPT); Test of Neuropsychological Malingering (TNM); Trail Making Test (TMT A-B); Digit Symbol Substitution Test (DSST); Verbal Fluency test. Administration: before and after shifts.	Cognitive test scores: no differences between the pretest and the post-test scores for both the 12- and 18-h duty schedules. Delay memory score: significant improvement between pre- and post-shift Depression scores: no differences between pretest and post-test scores for either the 12- or the 18-h duty schedules. Any cognitive test: no differences for the median percent change scores between 12- and 18-h schedules.	Satisfactory	Thomas et al., 2006 [30]
Innsbrusk, Austria	23 anaesthetists: 11 senior (9 M; 2 F; mean age = 49 ± 2 years) and 12 trainees (7 M; 5 F; mean age = 29.7 ± 1 years).	Length of shifts: 24 h.	Recognition reaction time; Motor reaction time; Critical flicker fusion; Response measure; Peripheral awareness task—recognition time; Visual analog scale (VAS); Maslach Burnout Inventory (MBI-D). Administration: at the beginning and after 24 h of in-house duty (8-h day shift and 16-h on-call duty).	No significant differences in pre- or post- service assessment between the two age groups. Reaction time: a trend towards a prolonged time in the pre- and post-service evaluation in senior anesthetists.	Satisfactory	Lederer et al., 2006 [58]
Zagreb, Croatia	26 anesthesiology residents (6 M; mean age = 29.9 years).	Length of shifts: 24 h.	Digit Symbol Substitution Test (DSST); Auditory Verbal Learning Test (AVLT); Circadian Type Inventory. The Stanford Sleepiness Scale Administration: at the beginning of the shift (8:00), and at the end of the shift (8:00 on the next day). The Circadian type Questionnaire in the pre-shift testing only.	AVLT: The magnitude of the total number of words recalled on the 5th and 6th trials was positively correlated with the age of subjects only after the shift. Digit span: the ability to concentrate did not change after the shift, while the working memory scores were increased significantly.	Unsatisfactory	Tadinac et al., 2014 [53]
Canada	28 Nurses (mean age = 39 years). Case group working 12-hr rotations (*n* = 14) Control group working 8-hr rotations (*n* = 14).	Work shift schedule: 4 working days followed by several rest days. Length of shifts: 12 h for one group and 8 h for another group.	Karolinska Sleeping Scale (KSS); Workplace Cognitive Failure Scale. Administration: start on the first free evening before the start of the working week and continued until the last day of working hours for 4 consecutive shifts.	Cognitive errors: no differences in cognitive errors were reported for nurses working 12-hr rotations compared to controls. No main effects of shift patterns on the memory, attention and action subscales.	Satisfactory	Rhèaume et al., 2018 [59]
Vienna, Austria	34 physicians (8 F, 26 M; mean age = 42.1 ± 8.6 years).	Work shift schedule: 08–16 for regular day shift (condition 1); 08–08 the next day for day-night shift (condition 2). Length of shifts: 8 h for condition 1 and 24 h for condition 2.	Sleep questionnaire form A (SF-A); Mood questionnaire (MDBF); Morning- evening questionnaire German version (D- MEQ); Visual memory test (FVW). Administration: before and at the end of shifts.	Significant reduction of post-duty mental state based on MDBF in all three dimensions mood, vigilance, agitation after the night shift. Significantly more errors in visual recognition after the night shift. No differences in the number of correctly identified elements and in the average reaction time.	Good	Osterode et al., 2018 [54]
South Africa	29 Anaesthesiology trainees (14 M; 15 F; mean age = 33 years).	Length shifts: 14-h night shift.	CogStateTM computerized test battery Administration: after the completion of the 14-h night shift, which ran from 17:00 to 07:00.	Significant reduction in response speed from pre to post-call testing in detection and identification tests. Absolute mean decline in the speed of the one card learning and one back speed tests but not significant No significant differences in the accuracy for the four tests administered before and after the call.	Good	Adams and Venter 2020 [55]
USA	308 Workers (159 M; 149 F; mean age = 74.07 ± 5.45 years).	Two groups based on working hours: ≥40 h of work per week (long working hours) and <40 h of work per week (short/normal working hours).	Three cognitive tests measuring: Orientation test Memory test; Executive functioning test.	Cognitive performance: older adults (≥40 h/week) were 1.76 times more likely to have a decreased performance compared to those engaged <40 h/week and.	Very good	Sagherian and Rose 2020 [57]
South Korea	352 firefighters (328 M; 24 F; mean age = 40.1 ± 8.7)	Work shift schedule: 3-day cycle (a full day (24 h) of work, followed by 2 days off-duty); 6-day cycle: 2 days of daytime work, followed by 2 days of nighttime work and 2 days off-duty; 9-day cycle: 3 days of daytime work, followed by 3 consecutive sets of nighttime work and off-duty; 21-day cycle: 5 days of daytime work, followed by 2 days off-duty, 3 consecutive sets of night time work and off-duty.	Central nervous system vital signs (CNSVS). Administration: during day work and the next day after night work.	CNSVS: decrease in neurocognitive scores the next day after nighttime work in composite memory (84.7 ± 19.7), verbal memory (81.3 ± 21.9), visual memory (94.0 ± 16.6), complex attention (93.3 ± 32.4), psychomotor speed (110.1 ± 15.2), motor speed (108.7 ± 14.1), neurocognitive index (97.4 ± 13.4) compared to the scores obtained during the daytime work (90.6 ± 19.1, 87.7 ± 20.0, 97.1 ± 16.3, 97.8 ± 18.2, 112.4 ± 15.4, 111.0 ± 15.1, in the previous domains, respectively).	Very good	Kwak et al., 2020 [26]
Poland	Shift workers: 18 paramedics (12 M; 6 F; mean age = 31.83 ± 4.73 years; 16 M firefighters (mean age = 33.19 ± 5.47 years); Control group: 17 persons (15 M; 2 F; mean age = 33 ± 4.32 years) working during the day	Work shift schedule: 12-h shifts (day and night shifts) and 24-h shifts with a 48- h break from work. Length of shifts: 12 and 24 h.	Attention network test (ANT); N -back task. Administration: 3 times for each group. Depending on the group, functioning after a night shift (paramedics)/24-h shift (firefighters), a day shift (paramedics, control group), a day off in the morning (firefighters, control group) and a day off in the evening (all groups).	Reaction time for the N-back task or in answer correctness: no significant differences between the groups on day off. Reaction time to a matched and non-matched stimulus: the control group had much longer and shorter reaction times than the group of paramedics when responding to a non-matched and matched stimulus, respectively. ANT analysis: significantly longer reaction times and significantly higher answer correctness in paramedics and firefighters compared to controls.	Satisfactory	Suminska et al. 2020 [56]

## Data Availability

Not applicable.

## References

[1] Benjafield J.G., Smilek D., Kingstone A. (2010). Cognition.

[2] Harvey P.D. (2019). Domains of cognition and their assessment. Dialogues Clin. Neurosci..

[3] Mesulam M. (2000). Principles of Behavioral and Cognitive Neurology.

[4] Lezak M.D., Howieson D.B., Loring D.W. (2004). Neuropsychological Assessment.

[5] Sachdev P.S., Blacker D., Blazer D.G., Ganguli M., Jeste D.V., Paulsen J.S., Petersen R.C. (2014). Classifying neurocognitive disorders: The DSM-5 approach. Nat. Rev. Neurol..

[6] Kielhofner G. (2009). Conceptual Foundations of Occupational Therapy Practice.

[7] Ansiau D., Wild P., Niezborala M., Rouch I., Marquié J.C. (2008). Effects of working conditions and sleep of the previous day on cognitive performance. Appl. Ergon..

[8] Kazemi R., Haidarimoghadam R., Motamedzadeh M., Golmohamadi R., Soltanian A., Zoghipaydar M.R. (2016). Effects of Shift Work on Cognitive Performance, Sleep Quality, and Sleepiness among Petrochemical Control Room Operators. J. Circadian Rhythms..

[9] Tucker-Drob E.M. (2011). Neurocognitive functions and everyday functions change together in old age. Neuropsychology.

[10] Salthouse T.A., Ferrer-Caja E. (2003). What needs to be explained to account for age-related effects on multiple cognitive variables?. Psychol. Aging.

[11] Salthouse T.A. (2010). Major Issues in Cognitive Aging.

[12] van der Flier W.M., Pijnenburg Y.A., Schoonenboom S.N., Dik M.G., Blankenstein M.A., Scheltens P. (2008). Distribution of APOE genotypes in a memory clinic cohort. Dement. Geriatr. Cogn. Disord..

[13] Jessen F., Wolfsgruber S., Wiese B., Bickel H., Mösch E., Kaduszkiewicz H., Pentzek M., Riedel-Heller S.G., Luck T., Fuchs A. (2014). German Study on Aging, Cognition and Dementia in Primary Care Patients. AD dementia risk in late MCI, in early MCI, and in subjective memory impairment. Alzheimers Dement..

[14] Wang X., Wang H., Li H., Li T., Yu X. (2014). Frequency of the apolipoprotein E ε4 allele in a memory clinic cohort in Beijing: A naturalistic descriptive study. PLoS ONE.

[15] Robinson A.C., Davidson Y.S., Roncaroli F., Minshull J., Tinkler P., Horan M.A., Payton A., Pendleton N., Mann D.M.A. (2020). Influence of APOE Genotype on Mortality and Cognitive Impairment. J. Alzheimers Dis. Rep..

[16] Cho H., Kim Y.E., Chae W., Kim K.W., Kim J.W., Kim H.J., Na D.L., Ki C.S., Seo S.W. (2020). Distribution and clinical impact of apolipoprotein E4 in subjective memory impairment and early mild cognitive impairment. Sci. Rep..

[17] Baumgart M., Snyder H.M., Carrillo M.C., Fazio S., Kim H., Johns H. (2015). Summary of the evidence on modifiable risk factors for cognitive decline and dementia: A population-based perspective. Alzheimers Dement..

[18] Peters R., Booth A., Rockwood K., Peters J., D’ Este C., Anstey K.J. (2019). Combining modifiable risk factors and risk of dementia: A systematic review and meta-analysis. BMJ Open..

[19] da Silva R.A. (2015). Sleep disturbances and mild cognitive impairment: A review. Sleep Sci..

[20] Torossian M., Fiske S.M., Jacelon C.S. (2021). Sleep, Mild Cognitive Impairment, and Interventions for Sleep Improvement: An Integrative Review. West. J. Nurs. Res..

[21] Kazemi R., Motamedzade M., Golmohammadi R., Mokarami H., Hemmatjo R., Heidarimoghadam R. (2018). Field Study of Effects of Night Shifts on Cognitive Performance, Salivary Melatonin, and Sleep. Saf. Health Work.

[22] International Labour Organization (ILO) (1990). C171—Night Work Convention.

[23] Jehan S., Zizi F., Pandi-Perumal S.R., Myers A.K., Auguste E., Jean-Louis G., McFarlane S.I. (2017). Shift Work and Sleep: Medical Implications and Management. Sleep Med. Disord..

[24] Harrington J.M. (2001). Health effects of shift work and extended hours of work. Occup. Environ. Med..

[25] Esmaily A., Jambarsang S., Mohammadian F., Mehrparvar A.H. (2021). Effect of shift work on working memory, attention and response time in nurses. Int. J. Occup. Saf. Ergon..

[26] Kwak K., Kim B.K., Jang T.W., Sim C.S., Ahn Y.S., Choi K.S., Jeong K.S. (2020). Association between Shift Work and Neurocognitive Function among Firefighters in South Korea: A Prospective Before-After Study. Int. J. Environ. Res. Public Health..

[27] Bokenberger K., Ström P., Dahl Aslan A.K., Åkerstedt T., Pedersen N.L. (2017). Shift work and cognitive aging: A longitudinal study. Scand. J. Work Environ. Health.

[28] Leso V., Caturano A., Vetrani I., Iavicoli I. (2021). Shift or night shift work and dementia risk: A systematic review. Eur. Rev. Med. Pharmacol. Sci..

[29] Moher D., Liberati A., Tetzlaff J., Altman D.G., PRISMA Group (2009). Preferred reporting items for systematic reviews and meta-analyses: The PRISMA statement. PLoS Med..

[30] Thomas F., Hopkins R.O., Handrahan D.L., Walker J., Carpenter J. (2006). Sleep and cognitive performance of flight nurses after 12-h evening versus 18-hour shifts. Air. Med. J..

[31] Titova O.E., Lindberg E., Elmståhl S., Lind L., Schiöth H.B., Benedict C. (2016). Association between shift work history and performance on the trail making test in middle-aged and elderly humans: The EpiHealth study. Neurobiol. Aging.

[32] Saricaoğlu F., Akinci S.B., Gözaçan A., Güner B., Rezaki M., Aypar U. (2005). The effect of day and night shift working on the attention and anxiety levels of anesthesia residents. Turk. Psikiyatri. Derg..

[33] Machi M.S., Staum M., Callaway C.W., Moore C., Jeong K., Suyama J., Patterson P.D., Hostler D. (2012). The relationship between shift work, sleep, and cognition in career emergency physicians. Acad. Emerg. Med..

[34] Shwetha B.L., Sudhakar H.H. (2012). Influence of shift work on cognitive performance in male business process outsourcing employees. Indian J. Occup. Environ. Med..

[35] Shwetha B.L., Sudhakar H.H. (2014). Learning, Memory & Executive function in female BPO employees exposed to regular shifts. Natl. J. Physiol. Pharm. Pharmacol..

[36] Özdemir P.G., Selvi Y., Özkol H., Aydın A., Tülüce Y., Boysan M., Beşiroğlu L. (2013). The influence of shift work on cognitive functions and oxidative stress. Psychiatry Res..

[37] Kaliyaperumal D., Elango Y., Alagesan M., Santhanakrishanan I. (2017). Effects of Sleep Deprivation on the Cognitive Performance of Nurses Working in Shift. J. Clin. Diagn. Res..

[38] Petru R., Wittmann M., Nowak D., Birkholz B., Angerer P. (2005). Effects of working permanent night shifts and two shifts on cognitive and psychomotor performance. Int. Arch. Occup. Environ. Health.

[39] Thomas J., Overeem S., Dresler M., Kessels R.P.C., Claassen J.A.H.R. (2020). Shift-work-related sleep disruption and the risk of decline in cognitive function: The CRUISE Study. J. Sleep Res..

[40] Rollinson D.C., Rathlev N.K., Moss M., Killiany R., Sassower K.C., Auerbach S., Fish S.S. (2003). The effects of consecutive night shifts on neuropsychological performance of interns in the emergency department: A pilot study. Ann. Emerg. Med..

[41] Griffiths J.D., McCutcheont C., Silbert B.S., Maruff P. (2006). A prospective observational study of the effect of night duty on the cognitive function of anaesthetic registrars. Anaesth. Intensive Care..

[42] Chang Y.S., Wu Y.H., Hsu C.Y., Tang S.H., Yang L.L., Su S.F. (2011). Impairment of perceptual and motor abilities at the end of a night shift is greater in nurses working fast rotating shifts. Sleep Med..

[43] Chang Y.S., Chen H.L., Wu Y.H., Hsu C.Y., Liu C.K., Hsu C. (2014). Rotating night shifts too quickly may cause anxiety and decreased attentional performance, and impact prolactin levels during the subsequent day: A case control study. BMC Psychiatry.

[44] Haidarimoghadam R., Kazemi R., Motamedzadeh M., Golmohamadi R., Soltanian A., Zoghipaydar M.R. (2017). The effects of consecutive night shifts and shift length on cognitive performance and sleepiness: A field study. Int. J. Occup. Saf. Ergon..

[45] Ferguson S.A., Kennaway D.J., Baker A., Lamond N., Dawson D. (2012). Sleep and circadian rhythms in mining operators: Limited evidence of adaptation to night shifts. Appl. Ergon..

[46] Rouch I., Wild P., Ansiau D., Marquié J.C. (2005). Shiftwork experience, age and cognitive performance. Ergonomics.

[47] Marquié J.C., Tucker P., Folkard S., Gentil C., Ansiau D. (2015). Chronic effects of shift work on cognition: Findings from the VISAT longitudinal study. Occup. Environ. Med..

[48] Devore E.E., Grodstein F., Schernhammer E.S. (2013). Shift work and cognition in the Nurses’ Health Study. Am. J. Epidemiol..

[49] Weinmann T., Vetter C., Karch S., Nowak D., Radon K. (2018). Shift work and cognitive impairment in later life-results of a cross-sectional pilot study testing the feasibility of a large-scale epidemiologic investigation. BMC Public Health.

[50] Thomas J., Overeem S., Claassen J.A.H.R. (2019). Long-Term Occupational Sleep Loss and Post-Retirement Cognitive Decline or Dementia. Dement. Geriatr. Cogn. Disord..

[51] Thomas J., Ooms S.J., Mentink L.J., Booij J., Olde Rikkert M.G.M., Overeem S., Kessels R.P.C., Claassen J.A.H.R. (2020). Effects of long-term sleep disruption on cognitive function and brain amyloid-β burden: A case-control study. Alzheimers Res. Ther..

[52] Lockley S.W., Cronin J.W., Evans E.E., Cade B.E., Lee C.J., Landrigan C.P., Rothschild J.M., Katz J.T., Lilly C.M., Stone P.H. (2004). Harvard Work Hours, Health and Safety Group. Effect of reducing interns’ weekly work hours on sleep and attentional failures. N. Engl. J. Med..

[53] Tadinac M., Sekulić A., Hromatko I., Mazul-Sunko B., Ivancić R. (2014). Age and individual sleep characteristics affect cognitive performance in anesthesiology residents after a 24-hour shift. Acta Clin. Croat..

[54] Osterode W., Schranz S., Jordakieva G. (2018). Effects of night shift on the cognitive load of physicians and urinary steroid hormone profiles-A randomized crossover trial. Chronobiol. Int..

[55] Adams T.P., Venter S. (2020). All night long: An assessment of the cognitive effects of night shift work in anaesthesiology trainees. SAJAA.

[56] Sumińska S., Nowak K., Łukomska B., Cygan H.B. (2020). Cognitive functions of shift workers: Paramedics and firefighters-An electroencephalography study. Int. J. Occup. Saf. Ergon..

[57] Sagherian K., Rose K. (2020). Long work hours, prolonged daytime naps, and decreased cognitive performance in older adults. Chronobiol. Int..

[58] Lederer W., Kopp M., Hahn O., Kurzthaler I., Traweger C., Kinzl J., Benzer A. (2006). Post- duty psychomotor performance in young and senior anaesthetists. Eur. J. Anaesthesiol..

[59] Rhéaume A., Mullen J. (2018). The impact of long work hours and shift work on cognitive errors in nurses. J. Nurs. Manag..

[60] Pega F., Náfrádi B., Momen N.C., Ujita Y., Streicher K.N., Prüss-Üstün A.M., Descatha A., Driscoll T., Fischer F.M., Technical Advisory Group (2021). Global, regional, and national burdens of ischemic heart disease and stroke attributable to exposure to long working hours for 194 countries, 2000–2016: A systematic analysis from the WHO/ILO Joint Estimates of the Work-related Burden of Disease and Injury. Environ. Int..

[61] Eurofound (2017). Sixth European Working Conditions Survey–Overview Report (2017 Update).

[62] Moreno C.R.C., Marqueze E.C., Sargent C., Wright K.P., Ferguson S.A., Tucker P. (2019). Working Time Society consensus statements: Evidence-based effects of shift work on physical and mental health. Ind. Health.

[63] Nabe-Nielsen K., Garde A.H., Ishtiak-Ahmed K., Gyntelberg F., Mortensen E.L., Phung T.K.T., Rod N.H., Waldemar G., Westendorp R.G., Hansen Å.M. (2017). Shift work, long working hours, and later risk of dementia: A long-term follow-up of the Copenhagen Male Study. Scand. J. Work Environ. Health.

[64] Nabe-Nielsen K., Hansen Å.M., Ishtiak-Ahmed K., Grynderup M.B., Gyntelberg F., Islamoska S., Mortensen E.L., Phung T.K.T., Rod N.H., Waldemar G. (2019). Night shift work, long working hours and dementia: A longitudinal study of the Danish Work Environment Cohort Study. BMJ Open..

[65] Seidler A., Nienhaus A., Bernhardt T., Kauppinen T., Elo A.L., Frölich L. (2004). Psychosocial work factors and dementia. Occup. Environ. Med..

[66] Juda M., Vetter C., Roenneberg T. (2013). Chronotype modulates sleep duration, sleep quality, and social jet lag in shift-workers. J. Biol. Rhythms..

[67] Vetter C. (2020). Circadian disruption: What do we actually mean?. Eur. J. Neurosci..

[68] Vetter C., Fischer D., Matera J.L., Roenneberg T. (2015). Aligning work and circadian time in shift workers improves sleep and reduces circadian disruption. Curr. Biol..

[69] Chellappa S.L., Morris C.J., Scheer F.A.J.L. (2018). Daily circadian misalignment impairs human cognitive performance task-dependently. Sci. Rep..

[70] Weldemichael D.A., Grossberg G.T. (2010). Circadian rhythm disturbances in patients with Alzheimer’s disease: A review. Int. J. Alzheimers Dis..

[71] Moore R.Y., Silver R. (1998). Suprachiasmatic nucleus organization. Chronobiol. Int..

[72] Tranah G.J., Blackwell T., Stone K.L., Ancoli-Israel S., Paudel M.L., Ensrud K.E., Cauley J.A., Redline S., Hillier T.A., Cummings S.R. (2011). Circadian activity rhythms and risk of incident dementia and mild cognitive impairment in older women. Ann. Neurol..

[73] Schlosser Covell G.E., Dhawan P.S., Lee Iannotti J.K., Hoffman-Snyder C.R., Wellik K.E., Caselli R.J., Woodruff B.K., Wingerchuk D.M., Demaerschalk B.M. (2012). Disrupted daytime activity and altered sleep-wake patterns may predict transition to mild cognitive impairment or dementia: A critically appraised topic. Neurologist.

[74] Naismith S.L., Hickie I.B., Terpening Z., Rajaratnam S.M., Hodges J.R., Bolitho S., Rogers N.L., Lewis S.J. (2014). Circadian misalignment and sleep disruption in mild cognitive impairment. J. Alzheimer’s Dis..

[75] Ortiz-Tudela E., Martinez-Nicolas A., Díaz-Mardomingo C., García-Herranz S., Pereda-Pérez I., Valencia A., Peraita H., Venero C., Madrid J.A., Rol M.A. (2014). The characterization of biological rhythms in mild cognitive impairment. Biomed. Res. Int..

[76] Bjorvatn B., Stangenes K., Oyane N., Forberg K., Lowden A., Holsten F., Akerstedt T. (2006). Subjective and objective measures of adaptation and readaptation to night work on an oil rig in the North Sea. Sleep.

[77] Forberg K., Waage S., Moen B., Bjorvatn B. (2010). Subjective and objective sleep and sleepiness among tunnel workers in an extreme and isolated environment: 10-h shifts, 21-day working period, at 78 degrees north. Sleep Med..

[78] Durmer J.S., Dinges D.F. (2005). Neurocognitive consequences of sleep deprivation. Semin. Neurol..

[79] Terán-Pérez G.J., Ruiz-Contreras A.E., González-Robles R.O., Tarrago-Castellanos R., Mercadillo R.E., Jiménez-Anguiano A., Velázquez-Moctezuma J. (2012). Sleep deprivation affects working memory in low but not in high complexity for the N-back test. Neurosci. Med..

[80] Dumont M., Benhaberou-Brun D., Paquet J. (2001). Profile of 24-h light exposure and circadian phase of melatonin secretion in night workers. J. Biol. Rhythms.

[81] Gibbs M., Hampton S., Morgan L., Arendt J. (2002). Adaptation of the circadian rhythm of 6-sulphatoxymelatonin to a shift schedule of seven nights followed by seven days in offshore oil installation workers. Neurosci. Lett..

[82] Midwinter M.J., Arendt J. (1991). Adaptation of the melatonin rhythm in human subjects following night-shift work in Antarctica. Neurosci. Lett..

[83] Knauth P. (1996). Designing better shift systems. Appl. Ergon..

[84] Baulk S.D., Fletcher A., Kandelaars K.J., Dawson D., Roach G.D. (2009). A field study of sleep and fatigue in a regular rotating 12-h shift system. Appl. Ergon..

[85] Jia L., Du Y., Chu L., Zhang Z., Li F., Lyu D., Li Y., Li Y., Zhu M., Jiao H. (2020). Prevalence, risk factors, and management of dementia and mild cognitive impairment in adults aged 60 years or older in China: A cross-sectional study. Lancet Public Health.

